# Detection of *Acanthamoeba* from *Acanthamoeba* Keratitis Mouse Model Using *Acanthamoeba*-Specific Antibodies

**DOI:** 10.3390/microorganisms10091711

**Published:** 2022-08-25

**Authors:** Min-Jeong Kim, A-Jeong Ham, A-Young Park, Hae-Jin Sohn, Ho-Joon Shin, Fu-Shi Quan, Hyun-Hee Kong, Eun-Kyung Moon

**Affiliations:** 1 Department of Biomedical Science, Graduate School, Kyung Hee University, Seoul 02447, Korea; 2 Department of Microbiology, Ajou University School of Medicine, Suwon 16499, Korea; 3 Department of Medical Zoology, Kyung Hee University School of Medicine, Seoul 02447, Korea; 4 Medical Research Center for Bioreaction to Reactive Oxygen Species and Biomedical Science Institute, School of Medicine, Graduate School, Kyung Hee University, Seoul 02447, Korea; 5 Department of Parasitology, Dong-A University College of Medicine, Busan 49201, Korea

**Keywords:** *Acanthamoeba*, keratitis, diagnosis, antibody

## Abstract

Although the prevalence of *Acanthamoeba* keratitis (AK) is rare, its incidence in contact lens wearers has increased. *Acanthamoeba* infections can lead to the loss of vision if the diagnosis and treatment are delayed. In this study, we investigated the diagnostic potential of two antibodies raised against the adenylyl cyclase-associated protein (ACAP) and periplasmic binding protein (PBP) of *A. castellanii* in the AK mouse model. The specificity of ACAP and PBP antibodies to *Acanthamoeba* was confirmed by immunocytochemistry. AK mouse models were produced by corneal infections with *A. castellanii* trophozoites for 7 days and 21 days. Enzyme-linked immunosorbent assay results revealed that both ACAP and PBP antibodies successfully detected *Acanthamoeba* antigens in the tears and eyeball lysates of the AK mouse model. The detection levels of *Acanthamoeba* antigens were similar at both infection time points. Anti-*Acanthamoeba* IgG, IgA, and IgM antibodies were evaluated from the sera of the AK mouse model. Notably, IgM and IgA antibody responses were highest and lowest at both time points, respectively. Our findings revealed that both ACAP and PBP antibodies could detect *Acanthamoeba* antigens in the tears and eyeball lysates of the AK mouse model. These results provide important information for understanding *Acanthamoeba* infections and developing a new diagnostic tool for AK.

## 1. Introduction

*Acanthamoeba* keratitis (AK) is a severe infectious disease affecting the eyes that can result in corneal disorders or even permanent vision impairment if left untreated [1]. In many countries, AK frequently occurs in contact lens wearers with poor hygienic practices, such as rinsing the lenses with contaminated tap water [1,2,3]. AK is often misdiagnosed as keratitis of bacterial or fungal origin, as the clinical manifestations from infection with these microbial organisms are strikingly similar [4,5]. For accurate diagnosis of AK, several methods including culturing *Acanthamoeba* from corneal scrapings, confocal microscopy, polymerase chain reaction amplification, and histopathological examinations are employed [6,7]. Although *Acanthamoeba* culturing from corneal scrapes is considered to be the gold standard, this method inflicts immense pain during sample acquisition and takes several days to obtain the results [6,8].

*Acanthamoeba* spp. are ubiquitous organisms and consistent with this notion, serological surveys conducted in the past have reported that around 50% to 100% of healthy individuals possessed anti-*Acanthamoeba* antibodies [9,10]. However, these *Acanthamoeba*-specific antibodies have limited potential for AK diagnosis. Evidently, *Acanthamoeba*-specific antibodies were reported to be present in low quantities in patients suffering from AK [11]. Anti-*Acanthamoeba* IgA antibodies have been demonstrated to be essential for protection against AK, as they contribute to amoebic lysis by neutrophils [12,13,14]. Yet, immunoblotting results from two AK patients revealed that anti-*Acanthamoeba* IgA responses were comparable to those of control sera [15]. Given these circumstances, alternative strategies to enhance AK diagnosis are necessary. One potential strategy to improve antibody-based AK diagnosis would be developing a highly sensitive AK-specific antibody that targets the structural components of *Acanthamoeba* spp.

Early diagnosis is essential for effective treatment of AK and many studies on *Acanthamoeba*-specific antibodies have been conducted to improve the currently used diagnostic methods. Using a bacteriophage antibody display library, four antibody clones that specifically bind to *Acanthamoeba* spp. were identified [16]. Eight monoclonal antibodies (AMEC1-3, MTAC1-5) generated from immunized mice were reported to be capable of cyst binding, and one of them (MTAC5) was reported to also interact with trophozoites of *A. castellanii* clinical isolates [17]. Monoclonal antibody to a mannose-binding protein of *A. culbertsoni* inhibited the cytotoxicity of *A. culbertsoni* trophozoites in vitro [18,19]. Similarly, monoclonal antibodies targeting the galactose-binding protein of *Acanthamoeba* spp. also demonstrated protective effects against *A. castellanii* infection [20]. In our previous study, we produced five polyclonal antibodies against the inosine-uridine preferring nucleoside hydrolase (IPNH), chorismite mutase (CM), carboxylesterase (CE), adenylyl cyclase-associated protein (ACAP), and periplasmic binding protein (PBP) of *A. castellanii* and showed their diagnostic potential for AK [21,22,23,24,25]. Although these *Acanthamoeba*-specific antibodies could be useful for improving AK diagnosis, one major limitation of the aforementioned studies is the fact that these antibodies were primarily tested in vitro and have not been applied to in vivo studies. To this extent, evaluating the diagnostic potential of these specific antibodies in AK animal models would greatly contribute to improving their credibility as an AK diagnostic tool.

Here, we produced AK animal models by corneally inoculating *A. castellanii* in mice and evaluated the diagnostic potential of ACAP and PBP polyclonal antibodies against AK using murine tear samples and whole eye tissue lysates. Furthermore, we investigated the level of anti-*Acanthamoeba* IgG, IgM, and IgA in the sera of the AK mouse model by enzyme-linked immunosorbent assays.

## 2. Materials and Methods

### 2.1. Cell Culture

*Acanthamoeba castellanii* and human corneal epithelial (HCE) cells were obtained from the American Type Culture Collection (ATCC 30868 and ATCC PCS-700–010, respectively). *Fusarium solani* (NCCP 32678), *Pseudomonas aeruginosa* (NCCP 16091), and *Staphylococcus aureus* (NCCP 15920) were obtained from the Korea Centers for Disease Control and Prevention. *Acanthamoeba* trophozoites were cultured axenically in peptone-yeast-glucose (PYG) medium at 25 °C, and *Acanthamoeba* cysts were induced in encystment media (95 mM NaCl, 5 mM KCl, 8 mM MgSO_4_, 0.4 mM CaCl_2_, 1 mM NaHCO_3_ and 20 mM Tris-HCl, pH 9.0) at 25 °C for 2 days [26]. HCE cells were cultured at 37 °C with 5% CO_2_ in keratinocyte growth medium (KGM; Lonza, Portsmouth, NH, USA). *F. solani* was cultured in Sabouraud Dextrose (SD) media at 25 °C, and *P. aeruginosa* and *S. aureus* were cultured in Brain Heart Infusion (BHI) media at 37 °C.

### 2.2. Immunocytochemistry

HCE cells (3 × 10^5^ cells/well) were cultured on sterile cover glass in a 6-well plate with KGM at 37 °C with 5% CO_2_ for 24 h. The next day, *A. castellanii* trophozoites (5 × 10^5^ cells/well) or cysts (5 × 10^5^ cells/well) were added and co-cultured for 4 h. *F. solani*, *P. aeruginosa*, and *S. aureus* were cultured in the early exponential phase (OD_600nm_ = 0.8) and co-cultured with HCE cells and *A. castellanii* for 1 h. Cells were washed with ice-cold phosphate-buffered saline (PBS) and then fixed with 100% methanol (chilled at −20 °C) for 5 min. The cells were washed three times with PBS and permeabilized with PBST (0.2% Triton X-100 in PBS) for 5 min at room temperature (RT). Cells were blocked for 30 min at RT in blocking buffer (1% bovine serum albumin and 22.52 mg/mL glycine in PBST). Cells were incubated overnight at 4 °C with the ACAP or PBP antibodies in blocking buffer (1:200) and probed with CruzFluorTM 555 (CFL 555)-conjugated anti-rabbit IgG (1:400) (Santa Cruz, Dallas, TX, USA) for 2 h at RT. After washing three times with PBS, cells were stained with VECTASHIELD mounting medium (Abcam, Burlingame, CA, USA) and observed under a fluorescence microscope (Leica DMi8, Wetzlar, Germany).

### 2.3. Production of Acanthamoeba keratitis Mouse Model

A total of 19 eight-week-old female BALB/c mice were purchased from Orient Bio (Seongnam, Korea). AK mouse model was prepared as previously described [27]. Briefly, *A. castellanii* trophozoites (1 × 10^5^ cells) were incubated on the surface of the commercial soft contact lens (Proclear 1-day contact lenses, CooperVision, San Ramon, CA, USA) for 1 h. Scratches were made on the right eyeballs of all mice. After scratching the corneas of mice, contact lenses containing *Acanthamoeba* were gently placed on the eyeballs. The right eyelids were sutured and closed to ensure that the *Acanthamoeba*-incubated contact lens remain attached to the cornea. Mice were monitored daily for 21 days to assess AK development in the eyes. Sutures were opened at 7 and 21 days post-infection (dpi) (*n* = 7) for tear sample and eyeball collection. Tear samples were washed by putting 10 μL of ice-cold sterile PBS into each *Acanthamoeba*-inoculated eye and a total of 100 μL was collected. Whole eyeballs were homogenized in 500 μL of sterile PBS, and a cardiac puncture was performed for serum collection. All samples were immediately frozen at −80 °C until use. All experimental protocols involving animals were approved by the Institutional Animal Care and Use Committee of the Ajou University Medical Center (AUMC-IACUC-2021-0006), and all methods were processed according to the relevant guidelines and regulations.

### 2.4. Detection of Acanthamoeba Antigens Using ACAP and PBP Antibodies

To detect *Acanthamoeba* antigens from the tears and eyeball lysates of the AK mouse model, an enzyme-linked immunosorbent assay (ELISA) was performed using ACAP and PBP antibodies. Tears (0.5 μg) and eyeball lysates (0.5 μg) were coated with carbonate coating buffer in 96-well plates and then incubated overnight at 4 °C. The plate was washed three times with PBS containing 0.05% Tween 20 (PBST) and blocked with 0.2% gelatin in PBST at 37 °C for 1 h. ACAP and PBP antibodies diluted in PBS (1:250 for tears and 1:1250 for eyeball lysates) were added to the wells and incubated at 37 °C for 1 h. Horseradish peroxidase (HRP)-conjugated anti-rabbit IgG, IgA (Sigma-Aldrich, St. Louis, MO, USA), and HRP-conjugated anti-rabbit IgM (Southern Biotech, Birmingham, AL, USA) diluted 1:2000 in PBS were inoculated into each well and plates were incubated at 37 °C for 1 h. O-phenylenediamine (OPD) (San Francisco, CA, USA) substrate was dissolved in citrate-phosphate buffer (pH 5.0) containing H_2_O_2_. The optical density values at 450 nm were read using an EZ Read 400 microplate reader (Biochrom Ltd., Cambridge, UK).

### 2.5. Anti-Acanthamoeba Antibody Responses in AK Mouse Model

Anti-*Acanthamoeba* antibody responses were determined by ELISA. Anti-*Acanthamoeba* IgG, IgA, and IgM antibodies were detected from sera of the AK mouse model using *A. castellanii* trophozoites as antigens, with sera of naïve mice serving as a negative control. Briefly, 96-well plates were coated overnight at 4 °C with *Acanthamoeba* trophozoites (10 μg/mL) dissolved in carbonate coating buffer. Serum samples collected from AK mice (1:50 dilution in PBS) were added to respective wells. After incubating the plates at 37 °C for 1 h, HRP-conjugated anti-mouse IgG, IgA, and IgM (1:1000 dilution in PBS) were added and plates were incubated for 1 h. Substrate solution containing OPD and H_2_O_2_ was added to each well. Optical density readings at 450 nm were measured using EZ Read 400 microplate reader.

### 2.6. Statistical Analysis

All statistical analyses were performed using GraphPad Prism version 5 (San Diego, CA, USA). Data were expressed as mean ± SD. Statistical significance between the groups was determined by Student’s *t*-test and denoted using an asterisk. *p* values less than 0.05 was considered statistically significant (* *p* < 0.05, ** *p* < 0.01, and *** *p* < 0.001).

## 3. Results

### 3.1. Confirming the Specificities of ACAP and PBP Antibodies

**Figure 1:**
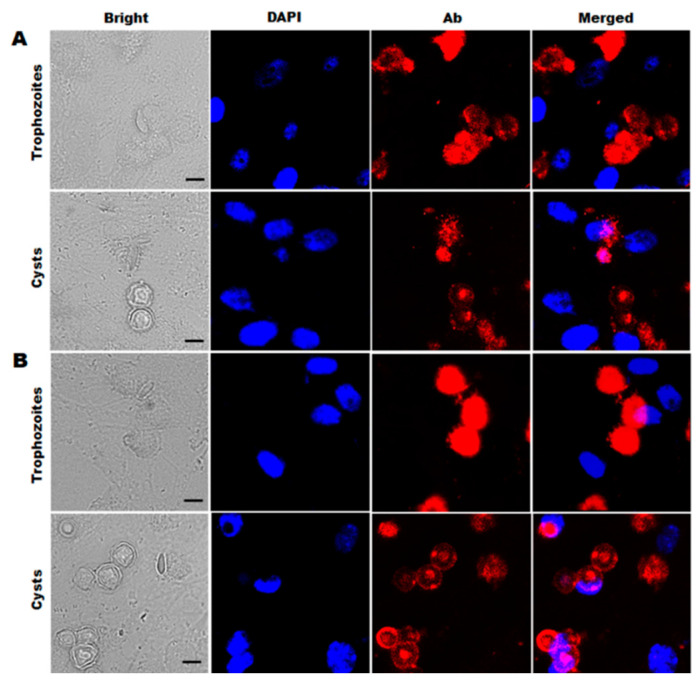
Specific detection of *Acanthamoeba* spp. by ACAP or PBP antibodies. Human corneal epithelial (HCE) cells were cultured with *A. castellanii* trophozoites or cysts, *F. solani*, *S. aureus*, and *P. aeruginosa*. The co-cultured cells were stained with DAPI and anti-ACAP antibodies (**A**) or DAPI and anti-PBP antibodies (**B**). Bright-field, DAPI staining (blue), ACAP or PBP antibodies combined with CFL555-conjugated secondary antibody (red), and merged images were acquired at 400× magnification. Scale bar = 10 μm.

To assess the diagnostic potential of the polyclonal antibodies against AK animal models, *Acanthamoeba* specificities of the ACAP and PBP antibodies were confirmed by immunocytochemistry. ACAP antibodies specifically detected the trophozoites and cysts of *A. castellanii* (red) from HCE cells co-cultured with *A. castellanii*, *F. solani*, *P. aeruginosa*, and *S. aureus* (Figure 1A). As expected, DAPI-stained nuclei (blue) of HCE cells and *F. solani* did not interact with the ACAP antibodies. Similar to the ACAP antibodies, *Acanthamoeba*-specific interaction was also confirmed from PBP antibodies. Strong immunoreactive signals were only detected from *A. castellanii* trophozoites and cysts microorganisms (Figure 1B). These results indicated that ACAP and PBP antibodies could specifically detect *A. castellanii* trophozoites and cysts, even when other microbial pathogens capable of causing corneal inflammation are present.

### 3.2. Production of AK Mouse Model

**Figure 2:**
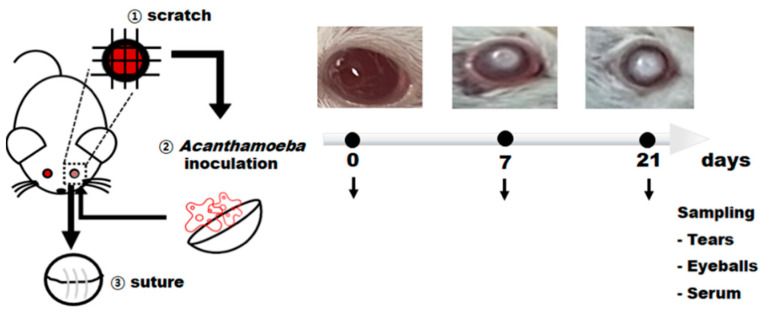
Experimental design for in vivo experiments to detect *Acanthamoeba* spp. from AK mouse model. BALB/c mice were infected with *A. castellanii* trophozoites for 7 days (*n* = 7) and 21 days (*n* = 7). Uninfected mice (naïve) were also run in parallel (*n* = 5). The tears and eyeball samples were collected from all groups were sacrificed at respective time points and used to detect *Acanthamoeba* antigens by ELISA. The collected sera were used to estimate anti-*Acanthamoeba* IgG, IgA, and IgM antibody responses.

To evaluate the antigen detection capability of ACAP and PBP antibodies, the AK mouse models at different time points (7 and 21 dpi) were produced. Animal model preparation and experiments were performed as illustrated (Figure 2). Corneas of mice were scratched for *A. castellanii* inoculation and subsequently sutured. At 7 and 21 dpi, a round corneal ulcer depicting AK was observed. Tear samples were acquired by washing the eyes with PBS as only a small number of tears could be collected.

### 3.3. Detection of Acanthamoeba Antigens from Tears of AK Mouse Models Using ACAP and PBP Antibodies

**Figure 3:**
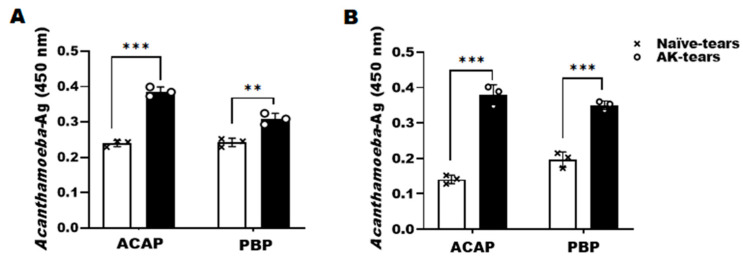
Detection of *Acanthamoeba* antigens in tears. The tears of the AK mouse model were collected after 7 (**A**) and 21 (**B**) dpi with *A. castellanii*. The detection of *Acanthamoeba* antigens in tears was evaluated by ACAP and PBP antibodies of *A. castellanii*. Negative (×); naïve tears, positive (○); AK-mouse tears. Data are expressed as mean ± SD and asterisks denote statistical significance between the means of the two groups (** *p* < 0.01 and *** *p* < 0.001).

ACAP and PBP antibodies of *A. castellanii* showed specific interactions with *Acanthamoeba* antigens in the tears of the AK mouse model. Interaction between these two antibodies with *Acanthamoeba* antigen elicited antibody responses that were significantly greater than those of naïve mice, irrespective of the infection time points. At 7 dpi, ACAP-induced antibody responses were higher than that of PBP antibody responses (Figure 3A). However, at 21 dpi, antibody responses between the two were quite similar (Figure 3B). Significant differences between the two antibodies at both time points were not observed.

### 3.4. Detection of Acanthamoeba Antigens from Eyeball Lysates of AK Mouse Models Using ACAP and PBP Antibodies

**Figure 4:**
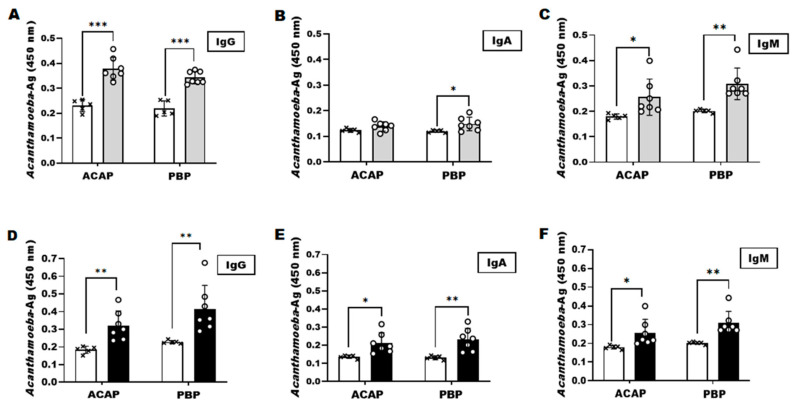
Detection of *Acanthamoeba* antigens in eyeball lysates. The eyeballs of the AK mouse model were collected after 7 (**A**–**C**) and 21 (**D**–**F**) dpi with *A. castellanii*. The eyeballs were sonicated in PBS and used for detection of the *Acanthamoeba* antigens by ACAP and PBP antibodies. IgG, IgA, and IgM antibody subtypes were used to determine the antigen-antibody responses. Negative (×); naïve eyeball lysates, positive (○); AK-mouse eyeball lysates. Data are expressed as mean ± SD and asterisks denote statistical significance between the means of the two groups (* *p* < 0.05, ** *p* < 0.01, and *** *p* < 0.001).

To confirm the presence of *Acanthamoeba* antigens in the corneas, mice were sacrificed, and their eyeballs were sampled. Consistent with the tear sample results, *Acanthamoeba*-specific antibody responses were detected from the eyeball lysates by both ACAP and PBP antibodies. At 7 dpi, antibody responses for the subtypes IgG, IgA, and IgM were strikingly similar for the two polyclonal antibodies (Figure 4A–C). However, by 21 dpi, PBP antibody-induced antibody responses were partially higher than those elicited by ACAP antibodies (Figure 4D–F). All antibody responses, except for the ACAP-induced IgA antibody at 7 dpi, were significantly greater than the naïve controls. Additionally, of the three antibody subtypes tested in this study, IgG antibody responses were the strongest while the weakest responses were observed from the IgA subtype. Combined, these results indicated that both ACAP and PBP antibodies are capable of detecting *Acanthamoeba* antigens in the tears and eyeball lysates of the AK mouse model.

### 3.5. Evaluation of Anti-Acanthamoeba Serum IgG, IgA, and IgM Antibodies in the AK Mouse Model

**Figure 5:**
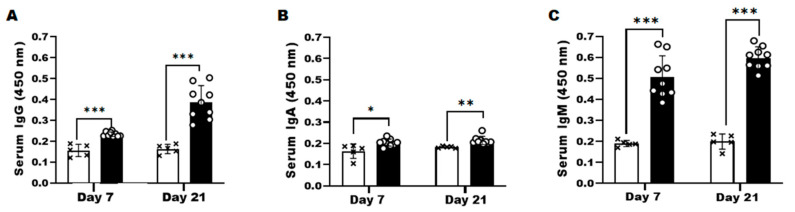
Evaluation of anti-*Acanthamoeba* IgG, IgA, and IgM antibodies in sera. The sera of the AK mouse model were collected after 7 and 21 dpi with *A. castellanii* and anti-*Acanthamoeba* serum IgG (**A**), IgA (**B**), and IgM (**C**) were observed by ELISA. Negative (×); naïve sera, positive (○); AK-mouse sera. Data are expressed as mean ± SD and asterisks denote statistical significance between the means of the two groups (* *p* < 0.05, ** *p* < 0.01, and *** *p* < 0.001).

To further confirm the presence of anti-*Acanthamoeba* serum IgG, IgA, and IgM antibodies in the AK mouse model, whole sera of mice at 7 and 21 dpi were acquired. While significantly higher than those of the naïve control group, *Acanthamoeba*-specific serum IgG and IgA responses were induced to a much lesser extent than the IgM subtype at 7 dpi. A noticeable increase in serum IgG responses was observed by 21 dpi, whereas IgA levels remained consistent. Surprisingly, IgM levels were maintained at high levels at all infection time points (Figure 5A–C). These findings indicated that of the three subtypes, *Acanthamoeba*-specific IgG and IgM are induced to a greater extent than the IgA subtype in infected mice.

## 4. Discussion

Direct detection of *Acanthamoeba* spp. in tears of AK patients could be a ground-breaking approach for the diagnosis of AK. In this study, we showed the detection of *Acanthamoeba* antigens in the tears and eyeball lysates of the AK mouse model using ACAP and PBP antibodies of *A. castellanii*. This is the first study reporting successful detection of *Acanthamoeba* antigens in the tears of AK mouse models using polyclonal *Acanthamoeba*-specific antibodies.

Currently, AK diagnosis in humans primarily relies on the patient’s medical history and initial clinical examination. However, AK can be misdiagnosed as keratitises of bacterial, fungal, or even viral origin as their symptoms are of non-specific nature [28]. To improve infectious disease diagnosis, immunological methods based on antigen or antibody detection from clinical specimens are actively being studied. Yet, serum anti-*Acanthamoeba* IgG and tear IgA antibody levels were reported to be induced at low levels in AK patients [11]. Given the current circumstances, developing highly sensitive *Acanthamoeba*-specific antibodies would enable rapid AK diagnosis. Recently, we have produced five specific antibodies to *Acanthamoeba* [21,22,23,24,25]. Among these antibodies, the potential application for AK diagnosis in vivo was demonstrated by ACAP and PBP antibodies and these were further evaluated to confirm our previous findings.

In the previous study, ACAP antibodies demonstrated *A. castellanii*-specificity to both trophozoite and cyst forms when these were co-cultured with HCE cells [24]. Here, we confirmed the specificity of ACAP antibodies to *A. castellanii* trophozoites and cysts co-cultured with HCE cells, *F. solani*, *S. aureus*, and *P. aeruginosa* (Figure 1A), thus signifying their potential for differential AK diagnosis. The specificity of the PBP antibodies to *A. castellanii* trophozoites co-cultured with HCE cells, *F. solani*, *S. aureus*, and *P. aeruginosa* has previously been reported [25], but their specificity to cysts remained unreported. Here, we confirmed that PBP antibodies are also capable of detecting the cysts of *A. castellanii* (Figure 1B). Combined, both ACAP and PBP antibodies are highly specific for *Acanthamoeba* and can be used to differentially diagnose AK, which makes them suitable for further in vivo studies. To confirm the *Acanthamoeba*-specificity of the antibodies reported in our previous in vitro studies, we produced an AK mouse model by corneal inoculation of *A. castellanii* trophozoites and demonstrated that both ACAP and PBP antibodies are capable of detecting *Acanthamoeba* antigens from the murine tears and eyeball lysates. While a total of 0.5 μg proteins acquired from the tears and eyeball lysates were used for detection, it is noteworthy to mention that the tears and eyeball lysates were diluted during the sampling procedure. Therefore, it is difficult to accurately discern exactly how much *Acanthamoeba* antigen is contained within these samples. However, in our previous study, we reported that ACAP antibodies could detect the presence of *Acanthamoeba* from cell lysate at concentrations as low as 0.01 μg [24]. Combining these two results, our findings suggest that the ACAP antibodies are highly sensitive to *Acanthamoeba* antigens and could be used to confirm the presence of *Acanthamoeba* even in trace amounts.

*Acanthamoeba* detection efficiency of antibody subtypes against eyeball lysates was assessed using indirect ELISA. Among the three secondary antibody subtypes tested in this study, the lowest response was observed from IgA. IgM responses were slightly greater than those of IgA but were less than those of IgG (Figure 4). While confirming these findings from tears would have been beneficial, this was extremely difficult to perform as only minuscule amounts of tear samples could be acquired from mice.

In addition to detecting *Acanthamoeba* antigen using *Acanthamoeba*-specific ACAP and PBP antibodies, serum antibody response to these antigens elicited upon *A. castellanii* infections were also assessed. Compared to the control group, antigen-specific IgM responses were highest at 7 dpi, whereas a marginal increase in IgG and IgA were observed at this time point. As expected, based on the antibody kinetics following infection, IgG levels were enhanced by 21 dpi. Interestingly, IgM responses were maintained at high levels even at 21 dpi. These antibody responses reported here are similar to those observed from the rabbit AK model, which reported significantly higher IgM induction than IgG [29]. Similar to the IgG profile reported in the present study, the anti-*Acanthamoeba* IgG antibody in rabbits peaked at 28 dpi. However, contrary to the rabbit IgM profile which peaked at 14 dpi and waned over time [29], such findings were not detected from the murine AK model. As the antibody kinetics appeared to somewhat differ between host organisms, evaluating the changes to antibody responses on a weekly basis could be beneficial.

There are several limitations of our study that must be taken into consideration. First and foremost, it is important to note that the timing of *Acanthamoeba* infection has a profound effect on the antibody-based detection demonstrated here. It is widely regarded that as time progresses, *Acanthamoeba* migrates from the surface into the deeper layers of the eye. At this stage, accurate AK diagnosis using the non-invasive antibody-based method described here could be difficult. Furthermore, there are several different species of *Acanthamoeba* that are capable of causing AK which include *A. castellanii*, *A. polyphaga*, *A. hachetti*, *A. culbertsoni*, *A. rhysodes*, *A. griffini*, *A. quina*, and *A. lugdunensis* [30]. Therefore, further studies should be warranted to investigate whether the antibodies described here are capable of detecting multiple members belonging to the genus *Acanthamoeba*. Additionally, alternative strategies that enable the detection of *Acanthamoeba* during the chronic phase of AK using these polyclonal antibodies should be investigated.

In summary, we have successfully demonstrated that both ACAP and PBP antibodies could be useful tools for diagnosing AK in mouse models. Since this method does not require corneal scrape culturing, the diagnosis time can also be greatly reduced. Although further confirmation of our antibodies using patient samples is required, the findings provided herein are promising and are expected to have a significant impact on the development of a quick and painless diagnosis for AK.

## Data Availability

Data supporting the findings of this study are contained within the article.
